# Melatonin Inhibits the Progression of Oral Squamous Cell Carcinoma *via* Inducing miR-25-5p Expression by Directly Targeting NEDD9

**DOI:** 10.3389/fonc.2020.543591

**Published:** 2020-12-02

**Authors:** Yanling Wang, Bo Tao, Jiaying Li, Xiaoqun Mao, Wei He, Qinbiao Chen

**Affiliations:** ^1^ Department of Stomatology, Henan Province Hospital of Traditional Chinese Medicine, Zhengzhou, China; ^2^ Department of Orthopedics, Tianjin Medical University General Hospital, Tianjin, China; ^3^ Huiqiao Medical Center, Southern Medical University Nanfang Hospital, Guangzhou, China; ^4^ Nursing Department, Sun Yat-Sen Memorial Hospital of Sun Yat-Sen University, Guangzhou, China; ^5^ Department of Oral and Maxillofacial Surgery, The First Affiliated Hospital of Zhengzhou University, Zhengzhou, China; ^6^ Neurosurgery Department, Sun Yat-Sen Memorial Hospital of Sun Yat-Sen University, Guangzhou, China

**Keywords:** melatonin, oral squamous cell carcinoma, miR-25-5p, anti-tumor, NEDD9

## Abstract

Melatonin exerts anti-cancer roles in various types of cancers. However, to the best of our knowledge, its role in oral squamous cell carcinoma (OSCC) is unknown. The present study aimed to investigate the role of melatonin and its underlying mechanism in OSCC. MTT, colony formation, wound healing, and transwell invasion assays proved that melatonin played anti-tumor effects in OSCC cells by inhibiting cell viability, proliferation, migration, and invasion in a concentration-dependent manner. The RT-qPCR analysis showed that miR-25-5p was significantly upregulated after melatonin treatment. Further, miR-25-5p might be involved in melatonin-induced inhibitory effects on the biological behavior of OSCC. The expression of miR-25-5p was decreased in tumor tissues and OSCC cells detected by RT-qPCR. MTT assay, colony formation assay, and TUNEL staining indicated miR-25-5p overexpression inhibited OSCC cell viability, proliferation, and induced OSCC cell apoptosis. Furthermore, wound healing, transwell invasion assay, and animal experiments suggested that miR-25-5p might exert suppressive effects on the migration, invasion, and tumor formation of OSCC cells, while miR-25-5p knockdown exhibited the opposite effects in OSCC cells. Bioinformatics analysis, western blot analysis, and luciferase reporter assay suggested that neural precursor cell expressed developmentally downregulated protein 9 (NEDD9) was proved to be a putative target for miR-25-5p. The role of NEDD9 in inhibiting OSCC cell proliferation, invasion, and migration was verified with NEDD9 siRNA transfection. Thus, melatonin exerted anti-proliferative, anti-invasive, and anti-migrative effects on OSCC *via* miR-25-5p/NEDD9 pathway. Melatonin could be applied as a potential novel drug on treating OSCC.

## Introduction

Oral cancer, as a global health problem, brings a huge challenge to the health care system. Oral squamous cell carcinoma (OSCC) occupies more than 90% of oral cancer (1). OSCC ranks as the 6th cancer type among the most common cancer types all over the world with a low 5-year overall survival rate and high incidence rate (2). It is estimated that there are 0.3 million new cases each year (3). Although considerable diagnostic and therapeutic progress has been made in recent years, the prognosis of the patients with OSCC remains particularly unfavorable because of its invasive characteristics and high malignancy (4). It is demonstrated that traditional treatments are not effective (5, 6). Thus, it is extremely urgent for us to widen the understanding of the mechanism underlying OSCC progression and identify novel and effective therapeutic methods.

Melatonin (N-acetyl-5-methoxytryptamine), a natural indoleamine, is mainly synthesized by the mammalian pineal gland and other tissues, such as lymphocytes, Harderian gland, liver, and gastrointestinal tract (7, 8). Interestingly, melatonin can regulate the circadian rhythms in living organisms, showing a wide distribution from bacteria to humans (9, 10). It has been shown that melatonin plays a vital role in the different physiological events, including the regulation of light/darkness responses, inhibition of tumor progression, improvement of immune system actions, and controlling of homeostasis in the different tissues (11–14). Besides, several pieces of evidence have revealed that melatonin could also serve as an antioxidant and oncostatic attributes (15, 16). According to the reports, melatonin exerts anti-cancer roles in various types of cancers, including breast cancer, lung cancer, colorectal cancer, gastric cancer, and cervical cancer (17–21). However, the underlying mechanism of the anti-cancer effects of melatonin on cancers needs further investigation.

Neural precursor cell expressed developmentally downregulated protein 9 (NEDD9) is a member of the Crk-associated substrate family. NEDD9 is located at 6p24.2 and also known as HEF1 and CasL. NEDD9 acts as a scaffold to regulate SRC and focal adhesion kinase pathways to modulate tumor cell adhesion, invasion, migration, proliferation, apoptosis, and survival (22–26). NEDD9 could activate multi-pathways, like PI3K/AKT, ERK, E-cadherin, Aurora-A (AURKA), and HDAC6. NEDD9 could also be activated by many stimuli, like TGF-β. At the end of mitosis, NEDD9 is degraded by proteasome. Although NEDD9 overexpression or inhibition does not induce tumorigenesis, its expression is upregulated in many cancers (27). NEDD9 could also regulate cancer metastasis. The upregulation of NEDD9 promotes multi cancer metastasis, like epithelial ovarian cancer, epithelial ovarian cancer, lung cancers, hepatocellular carcinoma, and cervical cancer (22, 28–34). NEDD9 could serve as a biomarker of tumor aggression and a prognostic gene of solid cancers. Further, NEDD9 could serve as one of the biomarkers for therapeutic resistance (27). Thus, NEDD9 might also regulate OSCC development.

MicroRNAs are a type of short (approximately 20~25 nucleotide), single-stranded non-coding RNAs, which are generally expressed in a diversity of tissues and cell types and mediate post-transcriptional gene silencing *via* binding to mRNA 3’UTR (35–37). Accumulating studies on the biological behaviors of miRNAs in the development, prognosis, proliferation, apoptosis, and differentiation have attracted the people’s attention (38). MicroRNAs (miRNAs) have been reported to exhibit a fundamental role in regulating a variety of physiological and pathological processes, including cancers (39). Recently, miRNAs, including miR-25-5p, have been shown to participate in the progression and metastasis of many cancers, including colorectal cancers (CRCs), non-small cell lung cancer (NSCL), and cutaneous squamous cell carcinoma (CSCC) (15, 40, 41). However, the expression, clinical significance, and functions of miR-25-5p in OSCC remain unclear. In the present study, we aimed to characterize the effects of melatonin on the development of OSCC and identify the underlying mechanisms.

## Materials and Methods

### Clinical Specimens

During the surgical procedure, the OSCC tissues (n=35) and adjacent tissues (n=35) were collected from patients with OSCC who had undergone surgical operation in Henan Province Hospital of TCM from January 2017 to October 2017 for research purpose. The patients with OSCC were diagnosed by histopathological analysis of tumor tissues from the surgical resection specimen. The specimen was examined and divided into OSCC tissues and adjacent tissues by faculties of the Pathology Department. Among the patients, a total of 35 patients, including 22 males and 13 females, were enrolled in this study. The age range was from 30 to 60 years old, with an average age of 41.5 ± 10.18 years. The clinical and pathologic characteristics of patients were obtained from the Medical Records Room. Patient information is shown in **Table 1**. All human tissues were snap frozen in liquid nitrogen and stored in a liquid nitrogen container (Thermo, USA) prior to further experiments. All the patients signed the informed consent before the study. The present study was approved by the Ethics Committee of Henan Province Hospital of TCM.

**Table 1:** The clinical characteristics of OSCC patients.

Characteristics	n	%
Age		
<41	19	54.29
≥41	16	45.71
Sex		
male	22	62.86
female	13	37.14
Tumor location		
Tongue	13	37.14
Gingival	8	22.86
Mouth floor	4	11.43
Lip	3	8.57
Cheek	4	11.43
Soft palate	3	8.57
Pathological differentiation grade		
Well	21	60.00
Moderate	11	31.43
Poor	3	8.57
Clinical stage		
I+II	19	55.47
III+IV	16	44.53

Clinical stage I: T1N0M0; Stage II: T2N0M0; Stage III: T3N0M0, T(1-3)N1M0; Stage IV: T4aN(0,1)M0, T(1-4a)N2M0, TN3M0, T4bNM0.

### Cell Culture

Human OSCC cells (SCC9) and the normal human oral keratinocytes (HOK) cells were purchased from the Biological Resources Center of ATCC, USA. The SCC9 cells were cultured in F12-Dulbecco’s modified Eagle’s medium (DMEM) culture medium supplemented with 10% fetal bovine serum (FBS) (Thermo, USA). The HOK cells were grown in DMEM culture medium with 10% FBS. All the cells were cultured in a 37°C, 5% CO_2_ humidified incubator (Thermo, USA). All cell lines were passaged for fewer than 6 months.

### MiRNA Transfection

The miR-25-5p mimic, miR-25-5p inhibitor or negative control (NC) mimic, and NC inhibitor used in this study were designed and synthesized by GenePharma, China. Human OSCC cells were transfected with miR-25-5p mimic or miR-25-5p inhibitor using the transfection reagent Lipofectamine2000 (Invitrogen, USA) according to the manufacturer’s instructions. The transfection concentration for either miR-25-5p mimic or NC mimic was 50 nM. The transfection concentration for either miR-25-5p inhibitor or NC inhibitor was 100 nM. Then, the cells were cultured for 48 h. RT-qPCR analysis was performed to confirm the transfection efficiency of miR-25-5p.

### Melatonin Treatment

Different concentrations (0 mM, 0.01 mM, 0.1 mM, and 1 mM) of melatonin (trans-3,5-dimethoxy-4-hydroxystilbene) (Selleckchem, USA) were added to the culture medium for 48 h to detect the effects of melatonin on the human OSCC cells.

### Real Time qPCR Analysis

RNA was extracted from tissues and cells using Trizol reagents (Invitrogen, USA) according to the manufacturer’s instructions. The concentrations and purification of RNAs were assessed by NanoDrop2000 spectrophotometer (Thermo, USA). For detecting the expression of miRNAs, a tissue/cell miRNA extraction kit (HaiGene, China) was used. The cDNAs were synthesized immediately from the RNAs to avoid RNA degradation using Reverse Transcription Kit (ABI, USA). The expression analysis of target genes was performed on Applied Biosystems StepOne Plus real-time PCR system (ABI, USA) by using TaqMan Universal PCR Master Mix (ABI, USA). The conditions were as follows: 95°C for 5 min, followed by 35~40 cycles of amplification (95°C for 30s, 60°C for 30s, and 72°C for 30s), and 72°C for 10 min. At last, the expression level of miRNA primers (forward and reverse) and U6 served as an endogenous control. GAPDH mRNA was used as an internal control to assess the relative expression of NEDD9 mRNA. The 2^-ΔΔCt^ method was utilized to detect the expression of target genes. In our study, the primers were designed and synthesized by GeenPharma, China. The primer sequences were shown in **Table 2**.

**Table 2:** The sequence of primers used for real-time qPCR analysis.

Genes	Primers sequences (5’ to 3’)
miR-21	Forward: GCTTATCAGACTGATGTTG
	Reverse: GAACATGTCTGCGTATCTC
miR-133a	Forward: TTTGGTCCCCTTCAACC
	Reverse: GAACATGTCTGCGTATCTC
miR-148a-3p	Forward: GTTCTGAGACACTCCGA
	Reverse: GAACATGTCTGCGTATCTC
miR-25-5p	Forward: CGGAGACTTGGGCAATT
	Reverse: GAACATGTCTGCGTATCTC
miR-155	Forward: TGCTAATCGTGATAGGGG
	Reverse: GAACATGTCTGCGTATCTC
U6	Forward: CTGACATCAGTGTCACAGACCC
	Reverse: CGCATCCTGTAGCAACTGTGTG
NEDD9	Forward: CCCATCCAGATACCAAAAGGACG
	Reverse: CACTGGAACTGAAAACACAGGGC
KLK9	Forward: TCAACCTCAGCCAGACCTGTGT
	Reverse: TCTCCAGGATGCTGATGTTGGC
WNT3A	Forward: ATGAACCGCCACAACAACGAGG
	Reverse: GTCCTTGAGGAAGTCACCGATG
FGF18	Forward: ACGATGTGAGCCGTAAGCAGCT
	Reverse: ACCGAAGGTGTCTGTCTCCACT
SRSF4	Forward: CAGATTAGTTGAAGACAAGCCAGG
	Reverse: CACTTCGGCTTCTGCTCTTACG
FIBP	Forward: CAAGGTGGTAGAGGAAATGCGG
	Reverse: CCTGTCTCAAAGCGGTTGTTAGC
SOX12	Forward: GACATGCACAACGCCGAGATCT
	Reverse: GTAATCCGCCATGTGCTTGAGC
TGFBI	Forward: GGACATGCTCACTATCAACGGG
	Reverse: CTGTGGACACATCAGACTCTGC
GAPDH	Forward: GTCTCCTCTGACTTCAACAGCG
	Reverse: ACCACCCTGTTGCTGTAGCCAA

### Methyl Thiazolyl Tetrazolium (MTT) Analysis

MTT assay was performed to investigate the cell viability of human OSCC cells. The cells were plated into 96-well plates. Then, 20 μL MTT solution (Biosharp, China) was added to each well. After incubation for 4 h, the MTT solution was discarded and 150 μL dimethyl sulfoxide (DMSO) was added. After incubation for additional 10 min, the absorbance at a wavelength of 490 nm were measured using a microplate reader (TECAN, Switzerland) to determine cell viability.

### Colony Formation Assay

Colony formation assay was performed to determine the proliferation ability of cells. First, 3500 cells were seeded into six-well plates (Corning, USA) and cultured for 14 days. The culture medium was replaced by free medium every three days. After three times washes with PBS, the cells were fixed with 4% paraformaldehyde (Solarbio, China) for 25 min at room temperature and stained with 0.2% crystal violet solution (Biosharp, China) for 20 min. The colonies (≥50 cells/colony) were observed and imaged under a light microscope (Nikon, Japan).

### Apoptosis Assay

Forty-eight hours after transfection, the cells were used for apoptosis assay. Transferase dUTP nick end labeling (TUNEL) assay was conducted using TUNEL staining kit (Ribo, China) according to the instructions. The TUNEL-positive cells were examined under a microscope (Nikon, Japan). The pictures from 10 random fields were observed and taken to assess the apoptosis of cells.

### Wound Healing Assay

Cell migration ability was examined by wound healing assay, and 5×10^5^ cells/well were plated into six-well plates (Corning, USA). When the density of cells reached about 90%, a wound was created at the bottom of plates using a sterile pipette tip. The cells were washed three times with PBS to clear cell debris and then cultured in the culture medium for 48 h. Finally, the images were captured under an inverted microscope (Nikon, Japan) at 0 h, 24 h, and 48 h.

### Transwell Invasion Assay

Cell invasion ability was tested by transwell invasion assay. For the transwell invasion assay, human OSCC cells were plated in the transwell chambers with 8 µm pore size polycarbonic membrane (Corning, USA) to separate the top chamber and the lower chamber. In brief, 1×10^5^ cells were seeded in serum-free DMEM in the upper chamber, which was coated with 20 µL extracellular matrix gel (Sigma, USA). The culture medium with 10% FBS was added into the lower chamber. After incubation for about 24 h, the cells on the top surface of the membrane were wiped off. The cells were then stained with crystal violet (Biosharp, China) at room temperature for 30 min. Finally, the cells were observed under a light and inverted microscope (Nikon, Japan).

### Animal Experiments

The animal experiments were performed with the approval of the Ethics Committee of Henan Province Hospital of TCM. Animal experiments were carried out according to the National Institutes of Health Guidelines to the Care and Use of Laboratory Animals. Forty Balb/c nude mice (4~6 weeks of age, male, Charles River, China) housed and maintained in a specific pathogen-free room, and were allowed free access to water and food. The mice were divided randomly into 4 groups (n=10 per group). To initiate OSCC xenografts, 5×10^6^ human OSCC cells transfected with miR-25-5p were injected subcutaneously into to the flanks of the nude mice. After 4 weeks, the animals were euthanized in a CO_2_ chamber and tumors were collected. Tumor nodules were collected and calculated by the following formula: V = (Width2 × Length)/2. The weights of tumors were weighed and analyzed.

### Bioinformatics Analysis

The candidate target genes of miR-25-5p were predicted using the TargetScanHuman 7.2 (http://www.targetscan.org/vert_72/) and miRWalk (http://mirwalk.umm.uni-heidelberg.de/). In Targetscan and miRWalk databases, the species was set as human. The miRNA was set as miR-25-5p. In Targetscan database, the predicted target genes of miR-25-5p was shown by searching the presence of conserved 8mer, 7mer, and 6mer sites that match the seed region of miR-25-5p. In miRWalk database, the interaction score of miR-25-5p and NEDD9 mRNA was 0.92. The binding sites of miR-25-5p and target genes’ mRNA 3’UTR were predicted and showed with TargetScan. NEDD9 could be predicted by both databases and might be a putative target for miR-25-5p.

### Western Blot Analysis

Human OSCC cells were lysed by using RIPA lysis buffer containing proteinase inhibitors (Beyotime, China). The concentration of proteins was detected according to the instruction of the BCA Protein Quantitation kit (Beyotime, China). Then, the total proteins (60 µg) were subjected to 10% sodium dodecyl sulfate (SDS)-polyacrylamide gel electrophoresis (PAGE) and transferred onto a PVDF membrane (Millipore, USA). Then, 5% skim milk was applied to block the membranes at room temperature for 120 min. Subsequently, immunoblotting was performed with specific antibodies against NEDD9 (1:1000, ab18056, Abcam, Cambridge, UK) and β-actin (1:2000, β-actin, Abcam, Cambridge, UK). β-actin was used as an internal control. The membranes were incubated with primary antibodies at 4°C temperature overnight. Next day, the secondary antibodies (Abcam, Cambridge, UK) were added and incubated at room temperature for 2 h. Ultimately, the signals were detected by an enhanced chemiluminescence (ECL) detection system (PerkinElmer, USA) and quantified with ImageJ software.

### Luciferase Reporter Assay

The 3’-UTR sequence of NEDD9 containing the predicted binding site for miR-25-5p was obtained and cloned into psiCHECK-2 vector (Promega, USA) to obtain the wild-type (WT) reporter plasmid NEDD9-WT. To generate the NEDD9 mutant (MUT) reporter plasmid, NEDD9-MUT, the seed region was mutated to eliminate all complementary nucleotides to miR-25-5p. Human OSCC were transfected with the reporter plasmid together with miR-25-5p mimic/miR-25-5p inhibitor and NEDD9-WT/NEDD9-MUT. After 48 h of transfection, a dual-luciferase reporter assay system (Promega, USA) was applied to monitor the relative luciferase activity.

### Statistical Analysis

All quantitative results were from at least three independent experiments and presented as the mean ± SD. Differences among various groups were evaluated by one-way analysis of variance (ANOVA) followed by Turkey’s post-hoc analysis. Diffeerences between two groups were analyzed with Student’s t-tests. All the statistical analysis was carried out using GraphPad Prism. A p-value of less than 0.05 was considered statistically significant.

## Results

### Anti-Tumor Effects of Melatonin on Human Oral Squamous Cell Carcinoma Cells

**Figure 1:**
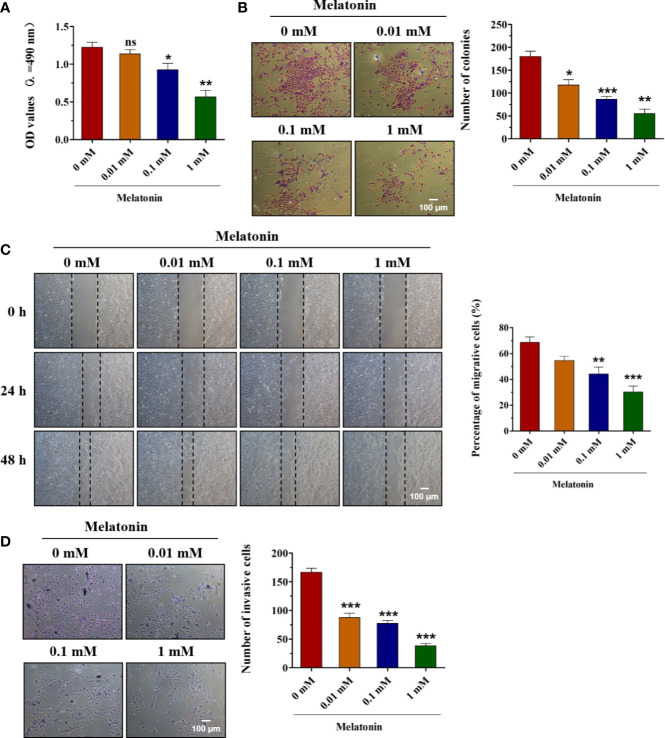
Effects of melatonin on the biological functions of OSCC cells. **(A)** SCC9 cells were treated with melatonin at 0.01, 0.1, and 1 mM for 48 h, and MTT assay was performed to detect the cell viability. **(B)** SCC9 cells were exposed to different concentrations of melatonin for 48 h and subjected to colony formation assay to examine the proliferation ability. **(C)** Effects of melatonin on the migration of SCC9 cells after exposure to melatonin (0.01, 0.1, and 1 mM) for wound healing assay. **(D)** The invasion ability of SCC9 cells after melatonin treatment by Transwell invasion assay. (n=4, One way ANOVA followed by the Tukey’s test, * indicated the differences compared with 0 mM Melatonin group, *p < 0.05, **p < 0.01, ***p < 0.001, ns, no statistical differences, Scale bar, 100 μm).

To test whether melatonin could exert the inhibitory effects in the biological functions of OSCC (SCC9) cells, various concentrations of melatonin were used. First, SCC9 cells were treated with 0.01, 0.1, and 1 mM of melatonin for 48 h. MTT assay indicated that, compared to control, melatonin decreased the cell viability of SCC9 cells in a concentration-dependent manner, and the maximum effect was 1 mM group (**Figure 1A**). The concentration-dependent effects of melatonin on the proliferation ability of SCC9 cells were also observed as confirmed by colony formation assay (**Figure 1B**). The number of colonies was significantly reduced by melatonin at concentrations of 0.01, 0.1, and 1 mM in SCC9 cells (**Figure 1B**). Wound healing assay showed that the number of migrating cells was reduced after melatonin treatment at 0.01, 0.1, and 1 mM, especially at 1 mM. These results suggested that melatonin showed a significant inhibitory effect on cell migration ability (**Figure 1C**). Dose-dependent inhibitory roles of melatonin in the invasion ability of SCC9 cells were displayed by the Transwell invasion assay (**Figure 1D**). These results suggested that melatonin played anti-tumor effects in SCC9 cells by inhibiting the cell viability, proliferation, migration, and invasion at a millimolar concentration.

### Melatonin Upregulates the Expression of miR-25-5p

**Figure 2:**
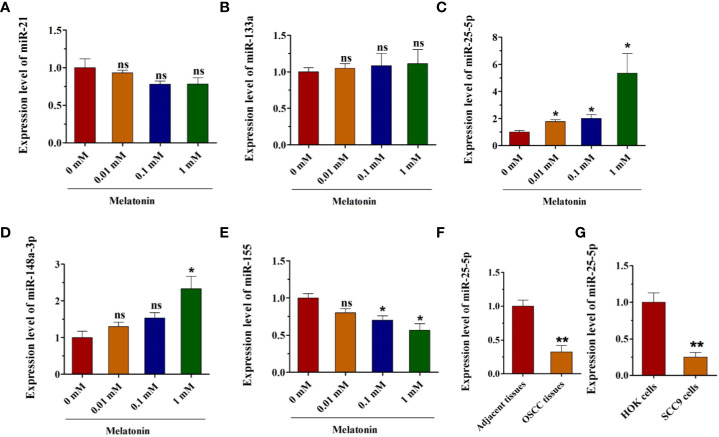
Melatonin increases the expression of miR-25-5p in OSCC cells. **(A–E)** RT-qPCR analysis of miR-21, miR-133a, miR-25-5p, miR-148a-3p, miR-155 expression was performed in SCC9 cells after treating with melatonin at 0.01, 0.1, and 1 mM for 48 h (n=4, One way ANOVA followed by the Tukey’s test, * indicated the differences compared with 0 mM Melatonin group). **(F)** Relative miR-25-5p levels in OSCC tissues (n=35, Student’s t-test). **(G)** RT-qPCR analysis was utilized to examine the expression of miR-25-5p in SCC9 and HOK cells (n=4, Student’s t-test) (*p < 0.05, **p < 0.01, ns, no statistical differences).

To understand the underlying mechanism of melatonin inhibiting the biological behaviors of OSCC cells, the RT-qPCR analysis was performed to identify the dysregulated expression of miRNAs in different concentrations of melatonin treated SCC9 cells. The results of RT-qPCR analysis revealed no significant changes in miR-21 or miR-133a expression after melatonin administration (**Figures 2A, B**). As shown in **Figures 2D, E**, melatonin elevated the expression of miR-148a-3p and miR-25-5p, but it inhibited the expression of miR-155 (**Figures 2C–E**). Among these miRNAs, miR-25-5p was the most significantly upregulated miRNA after melatonin treatment (**Figure 2C**). We hypothesized that miR-25-5p might be involved in the development of OSCC and the inhibitory effects of melatonin on OSCC cells. To further confirm this observation, the OSCC tissues and adjacent tissues were collected to detect the expression level of miR-25-5p. RT-qPCR assay based on 35 paired tumor tissues and matched tumor-adjacent tissues showed that miR-25-5p was significantly decreased in the OSCC tissues compared with the adjacent tissues (**Figure 2F**). In addition, as indicated in **Figure 2G**, the expression of miR-25-5p was markedly decreased in SCC9 cells compared with that in the normal human oral keratinocytes (HOK) cells (**Figure 2G**). These results suggested that miR-25-5p might be involved in melatonin-induced inhibitory effects on the biological functions of OSCC cells.

### Overexpression of miR-25-5p Inhibits Cell Viability, Proliferation, and Induces OSCC Cell Apoptosis

**Figure 3:**
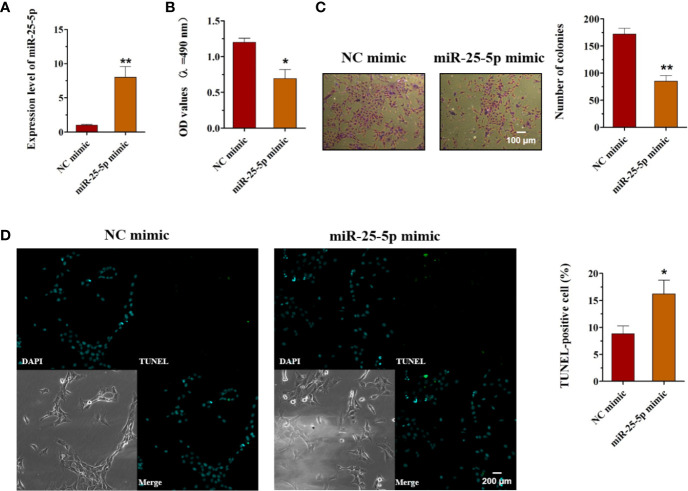
Overexpression of miR-25-5p inhibits the cell viability, proliferation but promotes the apoptosis of OSCC cells. **(A)** RT-qPCR analysis of miR-25-5p in SCC9 cells transfected with miR-25-5p mimic or NC mimic. **(B)** MTT assay of cell viability in SCC9 cells treated with miR-25-5p mimic or NC mimic. **(C)** Colony formation of SCC9 cells exposed to miR-25-5p mimic or NC mimic (Scale bar, 100 μm). **(D)** The apoptosis of SCC9 cells detected by TUNEL staining. Nuclei were stained by DAPI (blue) stain and apoptotic cells were stained by TUNEL (green) (Scale bar, 200 μm). (n=4, Student’s t-test, *p < 0.05, **p < 0.01).

As miR-25-5p was downregulated in OSCC, we hypothesized that miR-25-5p might serve as a tumor-suppressive miRNA in OSCC. To confirm this hypothesis, we transfected miR-25-5p mimics/mimics NC in OSCC cells. The expression levels of miR-25-5p in SCC9 cells were examined with RT-qPCR analysis 48 h after transfection. The results of RT-qPCR displayed that the miR-25-5p expression was significantly upregulated in SCC9 cells transfected with miR-25-5p mimic compared with that in the NC mimic group (**Figure 3A**). MTT assays showed that the cell viability of miR-25-5p mimic group was much lower than that in the NC mimic group (**Figure 3B**). The colony formation assay revealed that miR-25-5p mimic transfection significantly inhibited the proliferation ability compared with that in NC mimic-transfected cells (**Figure 3C**). TUNEL staining revealed that miR-25-5p overexpression led to an increase in apoptotic cell number in SCC9 cells (**Figure 3D**). These results supported the hypothesis that miR-25-5p inhibited the cell viability, proliferation, and induced the apoptosis of OSCC cells.

### Upregulation of miR-25-5p Suppresses the Migration, Invasion, and Tumor Formation of OSCC Cells

**Figure 4:**
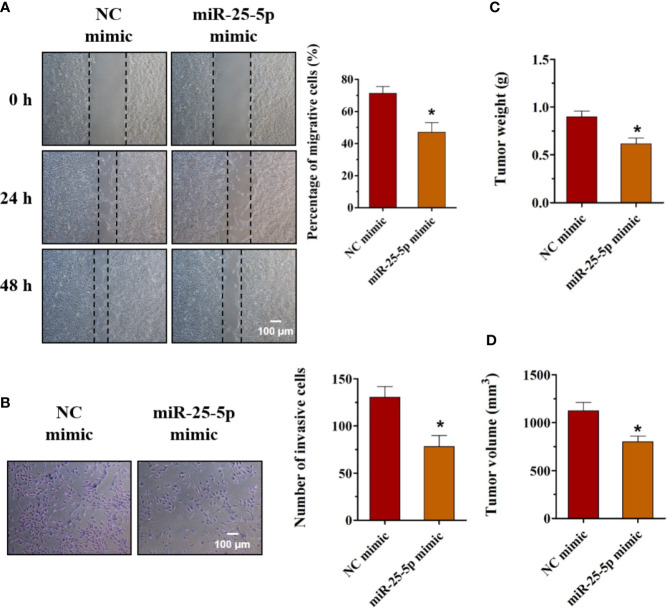
Upregulation of miR-25-5p suppresses the migration, invasion, and tumor formation of OSCC cells. **(A)** Wound healing assay was used to determine the migration of SCC9 cells transfected with miR-25-5p mimic or NC mimic (n=4). **(B)** SCC9 cells were transfected with miR-25-5p mimic or NC mimic and allowed to migrate through an 8 µm pore size polycarbonic membrane in Transwell chambers. The invasive cells were stained and counted (n=4, Scale bar, 100 μm). **(C, D)** Animal experiments of tumor formation of OSCC cells were conducted using SCC9 cells. Tumor weights and volumes were measured (n=10, Student’s t-test, *p < 0.05).

To unravel the function of miR-25-5p in OSCC cells, the oncogenic phenotypes, including migration, invasion, and tumor formation were detected. Wound healing assay showed that the SCC9 cells transfected with miR-25-5p mimic showed lower migratory capacity than the cells transfected with NC mimic, indicating that the increase of miR-25-5p led to a decrease in migratory ability of SCC9 cells (**Figure 4A**). The Transwell invasion analysis revealed that miR-25-5p overexpression was able to reduce the invasion of SCC9 cells (**Figure 4B**). To further determine the potential roles of miR-25-5p in OSCC tumor formation, animal experiments were performed. The SCC9 cells treated with miR-25-5p mimic or NC mimic were injected subcutaneously into the flanks of the nude mice. After 4 weeks, the tumors were collected and the tumor nodules were collected and calculated. As presented in **Figures 4C, D**, the tumor weights and tumor volumes in miR-25-5p mimic group were much lower than that in NC mimic group (**Figures 4C, D**). These data suggested that miR-25-5p might exert suppressive effects on the migration, invasion, and tumor formation of OSCC cells.

### Knockdown of miR-25-5 Promotes the Viability, Proliferation, and Inhibits the Apoptosis of OSCC Cells

**Figure 5:**
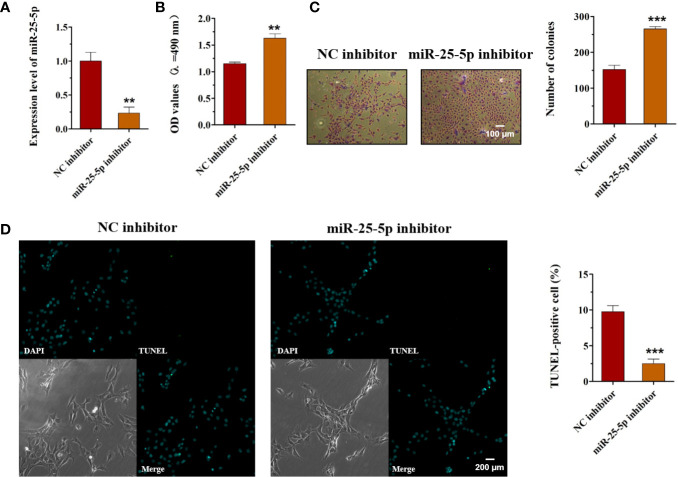
Knockdown of miR-25-5p promotes cell viability, proliferation, and inhibits the apoptosis of OSCC cells. **(A)** SCC9 cells transfected with miR-25-5p or NC inhibitor were used to analyze the expression of miR-25-5p with RT-qPCR. **(B)** MTT assay of cell viability in SCC9 cells in the presence of miR-25-5p or NC inhibitor. **(C)** Effect of miR-25-5p inhibitor on the proliferation ability of SCC9 cells (Scale bar, 100 μm). **(D)** The apoptosis of SCC9 cells detected by TUNEL staining. Nuclei were stained by DAPI (blue) stain and apoptotic cells were stained by TUNEL (green) (Scale bar, 200 μm). (n=4, Student’s t-test, **p < 0.01 ***p < 0.001).

To explore the role of miR-25-5p in OSCC, SCC9 cells were transfected with miR-25-5p inhibitor or NC inhibitor, and the cell viability, proliferation, and apoptosis were evaluated using MTT assay, colony formation assay, and TUNEL staining, respectively. First, the SCC9 cells were transfected with miR-25-5p inhibitor or NC inhibitor, and the expression of miR-25-5p was analyzed by RT-qPCR analysis (**Figure 5A**). As shown in **Figure 5A**, miR-25-5p inhibitor significantly inhibited miR-25-5p expression in SCC9 cells compared with NC inhibitor group (**Figure 5A**). Then, the transfected cells were selected for subsequent experiments. As shown in **Figure 5B**, knockdown of miR-25-5p markedly elevated the cell viability of SCC9 cells in comparison with the NC inhibitor group (**Figure 5B**). Furthermore, the number of colonies of SCC9 cells transfected with miR-25-5p inhibitor were significantly increased compared with SCC9 cells transfected with NC inhibitor (**Figure 5C**). Subsequently, TUNEL staining was utilized to determine the apoptosis of SCC9 cells. The results of TUNEL staining demonstrated that downregulation of miR-25-5p markedly suppressed the apoptosis of SCC9 cells compared with the NC inhibitor group (**Figure 5D**). The above results indicated that knockdown of miR-25-5p elevated the viability and proliferation but inhibited the apoptosis of OSCC cells.

### Knockdown of miR-25-5p Accelerates the Migration, Invasion, and Tumor Formation of OSCC Cells

**Figure 6:**
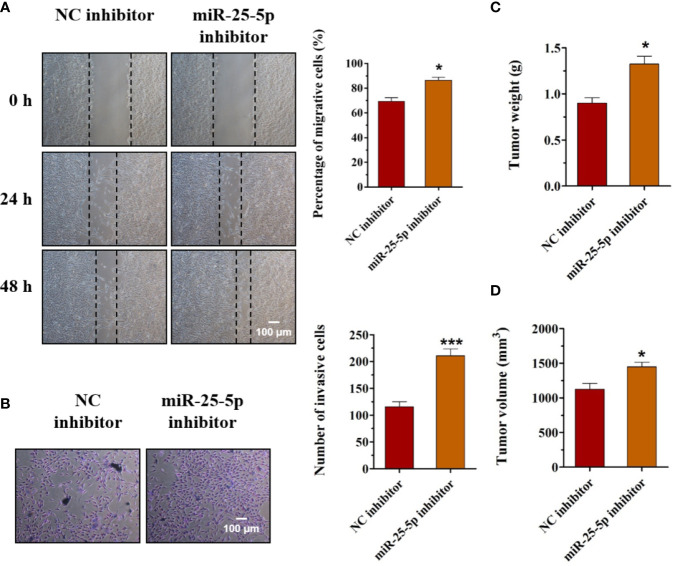
Knockdown of miR-25-5p accelerates the migration, invasion, and tumor formation of OSCC cells. **(A, B)** The effects of miR-25-5p inhibitor on the migration and invasion of SCC9 cells were detected by wound healing assay and transwell invasion assay, respectively (n=4). **(C, D)** The SCC9 cells transfected with miR-25-5p inhibitor or NC inhibitor were injected subcutaneously into the flanks of the nude mice. After 4 weeks, the tumor nodules were collected and the tumor formation was examined (n=10). (Student’s t-test, *p < 0.05 and ***p < 0.001, Scale bar, 100 μm).

To explore the functions of miR-25-5p in OSCC, we employed the miR-25-5p inhibitor and NC inhibitor in cultured SCC9 cells. The results of the wound healing and transwell invasion assays indicated that transfection of the miR-25-5p inhibitor obviously promoted both migration (**Figure 6A**) and invasion (**Figure 6B**) of SCC9 cells. Meanwhile, the knockdown of miR-25-5p increased the tumor weights and tumor volumes of Balb/c nude mice (**Figures 6C, D**). To conclude, the knockdown of miR-25-5p accelerated the migration, invasion, and tumor formation of OSCC cells.

### Inhibition of miR-25-5p Reverses the Inhibitory Effects of Melatonin in OSCC Cells

**Figure 7:**
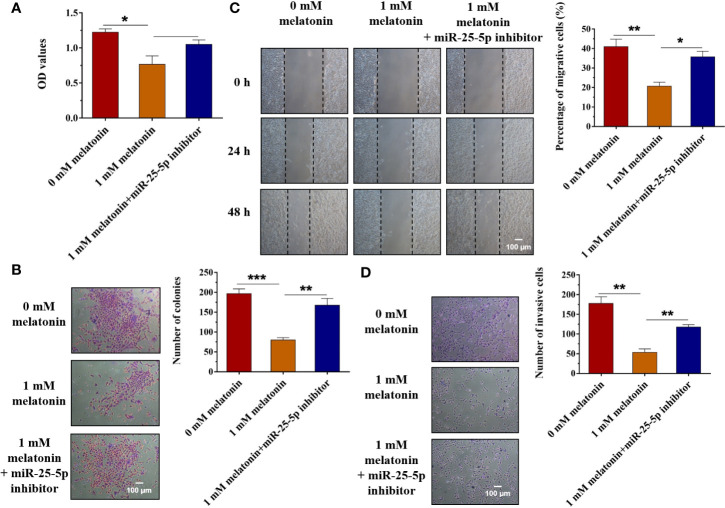
Inhibition of miR-25-5p reverses the inhibitory effects of melatonin in OSCC cells. **(A)** SCC9 cells were treated with melatonin at 1 mM and miR-25-5p inhibitor, and MTT assay was performed to detect the cell viability. **(B)** SCC9 cells were exposed to 1 mM melatonin and miR-25-5p inhibitor and then were subjected to colony formation assay to examine SCC9 cell proliferation ability. **(C)** Effects of melatonin on the migration of SCC9 cells in the presence of miR-25-5p by wound healing assay. **(D)** The invasion ability of SCC9 cells after melatonin treatment and miR-25-5p transfection by Transwell invasion assay. (n=4, One way ANOVA followed by the Tukey’s test, * indicated the differences compared with 0 mM Melatonin group,*p < 0.05, **p < 0.01, ***p < 0.001, Scale bar, 100 μm).

Then, we further investigated whether melatonin could exert the anti-proliferative, anti-invasive, and anti-migratory effects on OSCC cells by regulating the expression of miR-25-5p. According to the results of **Figure 1C**, the expression of miR-25-5p was the highest when the cells were treated with 1 mM melatonin. Therefore, the cells were treated with 1 mM melatonin and miR-25-5p inhibitor. MTT assay indicated that compared with the control group, the cell viability of OSCC cells were inhibited by 1 mM melatonin, while the inhibitory effects were abolished in the presence of miR-25-5p inhibitor (**Figure 7A**). As shown in **Figure 7B**, the number of colonies were markedly reduced by 1 mM melatonin, but it was increased in the OSCC cells treated with both 1 mM melatonin and miR-25-5p inhibitor (**Figure 7B**). Furthermore, the migratory ability of OSCC cells were obviously inhibited after 1 mM melatonin treatment. However, the inhibitory effects of melatonin on OSCC cell migration was offset by miR-25-5p inhibitor (**Figure 7C**). Transwell invasion analysis revealed that the inhibition of miR-25-5p could reverse the anti-invasive effects of melatonin in OSCC cells (**Figure 7D**). Above results indicated that melatonin exerted the anti-proliferative, anti-invasive, and anti-migratory effects on OSCC cells by regulating miR-25-5p.

### miR-25-5p Regulates OSCC Cell Proliferation, Invasion, and Migration *via* Targeting NEDD9

**Figure 8:**
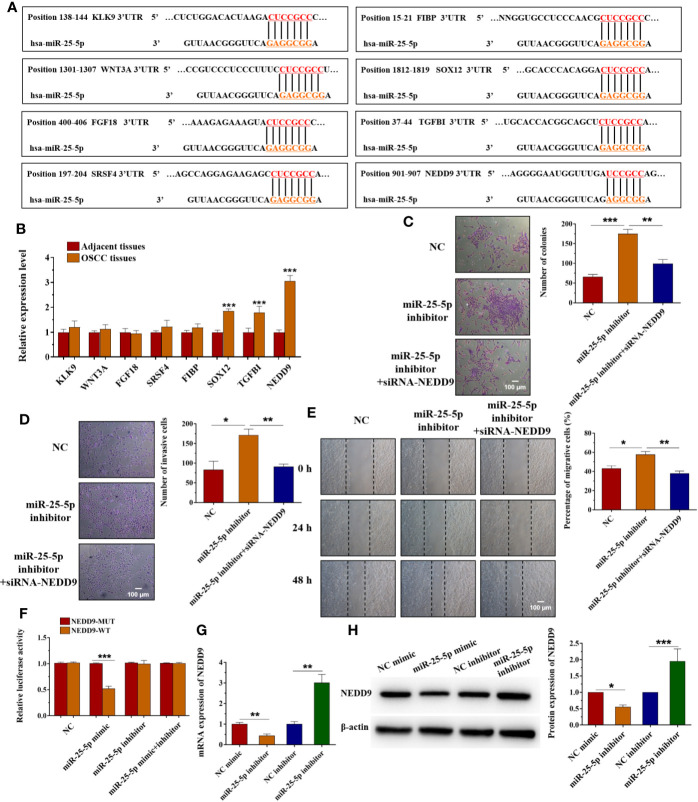
miR-25-5p regulates OSCC cell proliferation, invasion, and migration *via* targeting NEDD9. **(A)** Schematic representation of the predicted miR-25-5p binding region in the 3′-UTR of target genes. **(B)** The expression of KLK9, WNT3A, FGF18, SRSF4, FIBP, SOX12, TGFBI, and NEDD9 in OSCC tissues and adjacent tissues. **(C)** Colony formation assay was used to examine the proliferation ability. **(D)** Transwell invasion assay was performed to detect the invasion ability. **(E)** Wound healing assay was applied to assess the migration ability. **(F)** Luciferase activity was analyzed to confirm the relationship between miR-25-5p and NEDD9. **(G, H)** RT-qPCR **(G)** and western blotting **(H)** were performed to examine the NEDD9 level in SCC9 cells transfected with miR-25-5p mimic or NC mimic. (n=4, B: Student’s t-test; C-H: One way ANOVA followed by the Tukey’s test, * indicated the differences compared with NC mimics/inhibitor group, *p < 0.05, **p < 0.01, ***p < 0.001, Scale bar, 100 μm).

To determinate the mechanism underlying the effects of miR-25-5p in OSCC cells, the candidate target genes of miR-25-5p were predicted using the TargetScanHuman7.2 (http://www.targetscan.org/vert_72/) and miRWalk (http://mirwalk.umm.uni-heidelberg.de/). Among these target genes of miR-25-5p, KLK9, WNT3A, FGF18, SRSF4, FIBP, SOX12, TGFBI, and NEDD9 have been reported to participate in the development human cancers (42–49). Thus, we selected these genes for further investigation. The binding sequences between miR-25-5p and these target genes were shown in **Figure 8A**. The results of RT-qPCR indicated that among KLK9, WNT3A, FGF18, SRSF4, FIBP, SOX12, TGFBI, and NEDD9, the expression of NEDD9 was much higher in OSCC tissues than adjacent tissues. NEDD9 was the most obviously upregulated gene between OSCC tissues and adjacent tissues that isolated from OSCC patients (**Figure 8B**). Thus, we selected NEDD9 for further analysis. To further investigate the role of NEDD9, OSCC cells were transfected with miR-25-5p inhibitor, miR-25-5p inhibitor+siRNA-NEDD9 respectively. As shown in **Figure 8C**, compared with NC group, the proliferation ability of OSCC cells was elevated by miR-25-5p inhibitor, but it was inhibited in the presence of siRNA-NEDD9 (**Figure 8C**). The transwell assay showed that knockdown of miR-25-5p promoted the invasion ability of OSCC cells, which was reversed by siRNA-NEDD9 (**Figure 8D**). Furthermore, wound healing assay indicated that the migratory ability of OSCC cells in the miR-25-5p inhibitor group was higher than that in the NC group, while the elevated migratory ability was inhibited by NEDD9 siRNA (**Figure 8E**). To further confirm the relationship between miR-25-5p and NEDD9, dual-luciferase reporter assay was conducted. Luciferase reporter assay revealed that miR-25-5p mimic significantly decreased the luciferase activity in SCC9 cells transfected with NEDD9-WT, but not the NEDD9-MUT compared with NC group (**Figure 8F**). RT-qPCR analysis was conducted to determine the expression levels of NEDD9 in SCC9 cells after miR-25-5p modulation (**Figure 8G**). The results indicated that miR-25-5p mimic inhibited the mRNA expression of NEDD9, while miR-25-5p inhibitor elevated the mRNA expression of NEDD9 (**Figure 8G**). Western blot analysis demonstrated that miR-25-5p mimics reduced the protein level of NEDD9 and miR-25-5p inhibitor elevated the protein level of NEDD9 (**Figure 8H**). The results provided that NEDD9 was a direct target of miR-25-5p that regulated OSCC cell behaviors.

## Discussion

OSCC is one of the most malignant neoplasms worldwide and ranks first with 90% in oral cancers. There are about 0.3 billion new patients every year. Unhealthy living habits like smoking, alcohol uptake, and papillomavirus infection are the main risk factors of OSCC. With the progress of medical science in the recent decades, the 5-year survival rate of OSCC patients improves to approximate 50%. However, over 60% patients are at stage III or IV when diagnosed, which leaves a poor survival rate for these patients. It is necessary to clarify the mechanism of origination and development of OSCC, so as to find treatment targets and novel therapeutics (1–3).

Melatonin is an endogenous hormone secreted from pineal and could regulate circadian rhythms and mitochondrial homeostasis (12, 50, 51). Melatonin and its metabolites are proved to have an antioxidative role against oxidative stress (51). More interestingly, this hormone exerts anti-tumor effect on kinds of solid tumors *via* its receptor that exists in tumor tissues (13). It is thought that the anti-tumor effect of melatonin is based in its anti-oxidation and anti-inflammatory roles (52, 53). Melatonin inhibits triple negative breast cancer cell proliferation, migration *via* increasing miR-152-3p (54, 55). Melatonin inhibits breast tumor cell survival, migration, and invasion and upregulates miR-148a-3p (56). Melatonin represses 5-FU resistant colorectal cancer cell growth *via* miR-215-p/thymidylate synthase (TYMS) pathway (57). Melatonin inhibits gastric cancer cell growth *via* miR-16-5p/Smad3 pathway (58). Glioma cell proliferation and invasion are inhibited by melatonin *via* repressing miR-155 (59). In a random clinic trial, after neoadjuvant chemotherapy, melatonin was applied to treat OSCC patients. The residual tumor percentage and miR-210 were reduced. However, the decrease of miR-210 had no statistical significance (60). Thus, there could be other miRNAs downstream melatonin. After literature research, we chose miR-21, miR-133a, miR-148a-3p, miR-25-5p, and miR-155, which have been reported to regulate tumor progression in other cancers and have not been studied in OSCC. Among these miRNAs, miR-25-5p was downregulated in OSCC tissues and cells, and melatonin treatment upregulated miR-25-5p expression in OSCC cell. Further, we confirmed miR-25-5p was the downstream miRNA of melatonin in OSCC. Melatonin could inhibit multi tumor, like breast cancer, glioma, colorectal cancer, and gastric cancer. However, the involved pathways downstream melatonin in multi tumors are different.

MiRNAs, a cluster of noncoding RNAs, could regulate cell biological behaviors in tumors *via* targeting the 3’UTR of target genes (61). The role of miRNAs has been studied in multi tumors like breast and colon cancers (62, 63). In OSCC, the role of miRNAs has also been studied. Many miRNAs in the body fluids of OSCC patients are common, which indicated they could be set as biomarkers to predict diagnosis, prognosis, and therapeutic efficiency (64). MiRNAs are crucial therapeutic targets to handle oral cancer related pain (65). Thus, it might also be the targets to treat OSCC. According to the previous reports, miR-21, miR-133a, miR-148a-3p, miR-25-5p, and miR-155 participated in the development of various cancers. However, there were no studies that investigate the role of miR-21, miR-133a, miR-148a-3p, miR-25-5p, and miR-155 in OSCC cells and the relationship between melatonin and the expression of these miRNAs. Therefore, in our study, we performed RT-qPCR analysis to identify the dysregulated expression of miRNAs by using SCC9 cells, which were pretreated with melatonin at different concentrations. The results of RT-qPCR analysis revealed that no significant changes in miR-21 or miR-133a expression were observed under melatonin administration. As shown in the results, melatonin elevated the expression of miR-148a-3p and miR-25-5p, but it inhibited the expression of miR-155. Among these miRNAs, miR-25-5p was the most significantly upregulated miRNA after melatonin treatment. Therefore, we chose miR-25-5p for further analysis. MiR-25-5p has been reported to inhibit the proliferation of colorectal cancer cells (15, 66). In our study, melatonin upregulated the expression of miR-25-5p *in vitro*. Melatonin has been reported to upregulate lncRNA H19 *via* enhancing its transcription efficiency. H19 could target miR-675 to upregulate miR-675 expression (67). Thus, in this study, melatonin might upregulate miR-25-5p expression by promoting the expression of lncRNAs or transcription factors that binding to the promoter of miR-25-5p. This hypothesis needed further confirmation. The overexpression of miR-25-5p inhibited OSCC cell viability, proliferation, and induced cell apoptosis. The migration, invasion, and tumor formation were also inhibited by miR-25-5p. Our data confirmed the potential inhibitory role of miR-25-5p on OSCC. Besides, in our study, the results indicated that melatonin exerted anti-proliferative, anti-invasive, and anti-migratory effects on OSCC and promoted the expression of miR-25-5p. Further, inhibition of miR-25-5p could reverse the inhibitory effects of melatonin in OSCC cells. Therefore, we concluded that melatonin exerted anti-proliferative, anti-invasive, and anti-migratory effects on OSCC cells by regulating the expression of miR-25-5p.

As is widely known, miRNAs exert their posttranscriptional regulation role *via* inhibiting target genes expression. The potential target genes were predicted by two databases. There are lots of target genes of miR-25-5p according to the results of quick search on TargetScan databases. Among these target genes, KLK9, WNT3A, FGF18, FIBP, SOX12, TGFBI, and NEDD9 have been reported to participate in the development of human cancers (42–49). The results of RT-qPCR indicated that among KLK9, WNT3A, FGF18, SRSF4, FIBP, SOX12, TGFBI, and NEDD9, the expression of NEDD9 was much higher in OSCC tissues than adjacent tissues. NEDD9 displayed the most obviously upregulated gene between OSCC tissues and adjacent tissues that isolated from OSCC patients. Therefore, we speculated that NEDD9 might play a vital role in the development of OSCC. According to the results, NEDD9 might be the potential target gene of miR-25-5p, and we selected NEDD 9 for further analysis. Dual-luciferase reporter assay confirmed the interaction of miR-25-5p and NEDD9 mRNA. The mRNA expression of NEDD9 could be regulated by miR-25-5p. In OSCC cells, NEDD9 induced MMP9 secretion is an important process to form invadopodia (68). Abnormal expression of NEDD9 has been proved in colorectal cancer, lung cancer, and melanoma (69–71). The inhibition of NEDD9 could induce cancer cell apoptosis in colorectal cancer (15). NEDD9 could regulate many cellular behaviors like proliferation, invasion, mitosis, and migration (72). Overexpressed NEDD9 could enhance the metastasis of hepatocellular carcinoma, while inhibition of NEDD9 could suppress the metastasis (73).

In conclusion, our results proved miR-25-5p/NEDD9 was the downstream pathway of melatonin in OSCC. This study clarified a new mechanism and provided novel therapeutic targets in OSCC. Melatonin could be a potential treatment drug to handle OSCC.

## Data Availability Statement

All datasets generated for this study are included in the article/supplementary material.

## Ethics Statement

All the patients have signed the written informed consent before the study. And the present study was approved by the Research Ethics Committee of Henan Province Hospital of TCM. The animal experiments were performed with the approval of the Ethics Committee of Henan Province Hospital of TCM. Animal experiments were carried out according to the National Institutes of Health guidelines to the Care and Use of Laboratory Animals.

## Author Contributions

Authors WH and QC designed the study and wrote the protocol. Authors YW, BT, and JL conducted the experiments. Author BT managed the literature searches and analyses. Authors JL and XM undertook the statistical analysis, and author YW wrote the first draft of the manuscript. All authors contributed to the article and approved the submitted version.

## Funding

Funding for this study was provided by Henan Provincial Science and Technology Department Project (172102310613, 142300410086).

## Conflict of Interest

The authors declare that the research was conducted in the absence of any commercial or financial relationships that could be construed as a potential conflict of interest.
